# Proto-clinical optoretinography dependence on temporal and spatial resolution

**DOI:** 10.1364/BOE.596045

**Published:** 2026-06-10

**Authors:** Arman Athwal, Ringo Ng, Ayoub Faraji, Yifan Jian, Jem Love, Thomas Smart, Mohammad Shahidul Islam, Myeong Jin Ju, Marinko V. Sarunic

**Affiliations:** 1 Department of Medical Physics and Biomedical Engineering, University College London, London WC1E 6BT, United Kingdom; 2 Casey Eye Institute, Oregon Health & Science University, Portland, OR 97239, USA; 3 Department of Biomedical Engineering, Oregon Health & Science University, Portland, OR 97239, USA; 4 Institute of Ophthalmology, University College London, London EC1V 9EL, United Kingdom; 5 School of Biomedical Engineering, University of British Columbia, Vancouver, British Columbia V6T 1Z3, Canada; 6 Department of Ophthalmology & Visual Sciences, University of British Columbia, Vancouver, British Columbia V5Z 3N9, Canada

## Abstract

Optoretinography (ORG) enables non-invasive measurement of stimulus-evoked photoreceptor deformation using phase-sensitive optical coherence tomography (OCT). In this study, we evaluated how lateral resolution, acquisition speed, and spatial and inter-trial averaging influence velocity-based phase ORG measured with a raster-scanning spectral-domain OCT platform configured to resemble clinically realistic hardware. Seven healthy eyes were imaged while varying beam diameter (1.6–4.8 mm), B-scan rate (400–800 Hz), and averaging strategies. The characteristic biphasic response, a rapid contraction followed by slower elongation, was consistently observed across all conditions. Increasing NA improved structural cone visibility but did not proportionally enhance averaged ORG metrics, while moderate NA often yielded more stable signals. Higher acquisition speeds sharpened the measured contraction dynamics but reduced the signal-to-noise ratio due to increased phase noise. Both spatial and trial averaging substantially improved SNR. These findings demonstrate that repeatable phase-based ORGs can be achieved without cellular resolution or ultrahigh scan rates, and provide practical guidance for implementing proto-clinical ORG on conventional OCT systems.

## 1. Introduction

The retina is the multi-layered neural tissue at the back of the eye that captures incoming light and converts it into electrical signals which are passed to the brain, granting vision. At the core of this process are the photoreceptor cells (rods and cones) whose outer segments absorb photons and initiate phototransduction. While optical coherence tomography (OCT) has made visualization of the microscopic architecture of the retina routine in the clinic, enabling early diagnosis and monitoring of a wide range of diseases [1], assessment of the retina’s function is still largely confined to subjective tests like microperimetry or eye charts, which are not ideal as they rely on the patient’s feedback which can vary under different conditions. Meanwhile, the most common objective test in the clinical mainstream is electroretinography (ERG) which, although well-established and invaluable, is physically invasive and limited to measuring large regions of the retina at a time, and therefore cannot reveal local dysfunction that often precedes gross structural loss [2]. A technology that can deliver co-registered structural and functional information, non-invasively and with cellular-scale precision, would fill a critical gap in ophthalmic care.

Optoretinography (ORG) has emerged over the past decade as a compelling candidate for that role. Envisaged by Mulligan et al. in 1994 [3], ORG refers to the optical measurement of stimulus-evoked changes in the retina, obtained by coupling a visual-stimulus module to a high-resolution imaging system and analyzing either intensity or phase fluctuations in the back-scattered light. Intensity-based ORG, pioneered with adaptive-optics scanning laser ophthalmoscopy (AO-SLO), revealed that cone reflectance varies both spontaneously [4,5] and immediately after a flash [6]. Phase-based ORG, enabled by OCT, uses interferometric techniques to reference the phase of light returning from the inner-segment/outer-segment junction (IS/OS) to that from the photoreceptor tips, allowing for nanometre-scale length changes to be detected [7,8].

A succession of phase-ORG studies has characterized the standard biphasic photoreceptor response: a rapid ∼5 ms contraction (< 50 nm) followed by a slower ∼1 s elongation (> 100 nm). These signatures have been leveraged to classify cone spectral types [9], quantify rod versus cone signatures [10], and identify early dysfunction in retinal disease [11]. The systems used to pioneer ORG research have to date largely relied on advanced research hardware, including adaptive optics (AO) [12,13], line-scan [14] or full-field OCT [15,16], and high-speed acquisition systems [17]. These platforms are capable of resolving individual cones and producing exquisite functional maps, but their cost and complexity limit their potential for clinical translation.

Optoretinography with more clinically-friendly instrumentation have also been proposed. Vienola et al. combined point-scan OCT with a velocity-based metric that circumvents the need to track individual cones over seconds by analysing instantaneous phase slopes within 10 ms windows [18]. Meanwhile, Gong et al. showed that repeated-flicker stimulation combined with a 600 kHz raster-scan OCT could elicit robust photopic ORGs without dark adaptation [19]. These works are evidence of a growing interest in implementations of ORG that are less demanding technologically and logistically – ones that can be deployed in routine imaging rooms and yield robust, repeatable functional metrics across a wide patient population. However, how ORG signal quality depends on these parameters within clinically practical operating ranges has not been thoroughly characterised.

In this study, we evaluated the dependence of phase-based optoretinography on parameters that are commonly constrained in clinical OCT systems. In conventional imaging instruments, the transverse resolution is limited by the probe beam diameter entering the eye, which (along with the ocular length) determines the effective numerical aperture (NA) and therefore the focused spot size at the retina. While increasing the NA improves the transverse resolution, such configurations are often limited by optical aberrations by the refractive elements of the eye. Although prior studies have demonstrated improved structural visualization with larger beam diameters and adaptive optics, the impact of transverse resolution on phase-based ORG measurements using a non-AO raster-scanning OCT system has not been examined.

Specifically, we investigated the dependence of ORG signal quality on lateral resolution and B-scan rate within the ranges accessible on a clinical raster-scanning platform. We also examined the influence of scan averaging and intensity-based filtering on measured ORG metrics. To do so, we (i) adapted a custom spectral-domain OCT with a programmable projector for retinal stimulation, (ii) implemented a velocity-based ORG processing pipeline modeled after Vienola et al. [18] that operates on a standard workstation, and (iii) acquired repeated ORGs from healthy volunteers in under ten minutes per eye.

By systematically varying lateral resolution, scan speed, and averaging strategies, we identified acquisition regimes that preserve the familiar contraction and elongation features of the ORG response under conditions comparable to those of modern commercial OCT systems. Rather than pursuing research-grade imaging performance, this work evaluates whether clinically available hardware, when appropriately configured and processed, can generate repeatable ORG measurements. These findings provide practical guidance for implementing combined structural and functional retinal imaging without reliance on specialized research-grade instrumentation.

## 2. Methods

### 2.1. OCT optoretinography system

**Fig. 1.**
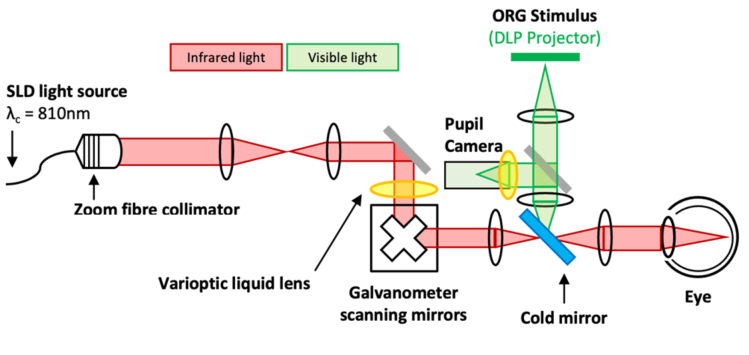
SD-OCT Sample arm diagram showing OCT and ORG beam paths.

The custom spectral-domain OCT system used for this study employed a superluminescent diode (SLD) light source of 810 nm centre wavelength and 100 nm bandwidth (BLM2-D-810-B-5; Superlum). The system was configured in a dual-spectrometer configuration [20]. For our experiments, we operated the system at A-scan rates of either 200 kHz (low speed) or 400 kHz (high speed). The system also featured a zoom collimator (ZC618APC-B; Thorlabs), permitting a tunable numerical aperture (NA) by varying the beam diameter incident on the cornea between 1.6 mm (low NA) – 4.8 mm (high NA). A Varioptic liquid lens (Corning) was used to correct for defocus, and the beam was relayed to the pupil via a pair of galvanometer scanners. The optical power for the OCT in the sample arm at the pupil was set to 0.50 mW. The imaged field of view was approximately 0.40 mm on the retina, corresponding to an angular extent of approximately 1.35° [21]. The sample arm configuration is illustrated in Fig. 1.

The ORG stimulus was delivered via a digital light processing projector (DLP LightCrafter 4500; Texas Instruments) co-aligned with the OCT beam via a cold mirror (FM03R; Thorlabs). The stimulus colour was set to green with a central wavelength of 540 nm and a full-width half-maximum of 80 nm. This bandwidth of light overlaps with the spectral sensitivities of L- and M-cones, which make up the majority of cones in the retina [22]. The stimulus was configured to be a uniform, 30 ms flash in the shape of a rectangle that was 1.2 mm × 0.40 mm on the retina, where the long axis (1.2 mm) is oriented along the B-scan direction and the short axis (0.40 mm) is perpendicular to it. The OCT B-scan covers approximately 0.40 mm on the retina, so the stimulus region is three times larger than the scanned line along the scan direction but matched to it in the perpendicular axis. The decision to stimulate a wider region than the imaged region was to allow for a tolerance in motion of the subject. In other words, even in the case of out-of-plane motion, the ORG signal is still collected from nearby cones that have been stimulated to an equivalent level of bleach. For all experiments, the optical power of the flash was 55 µW. To calculate percentage bleach of the stimulus, we followed Vienola et al., and given that the duration of the flash and the area over which it was delivered are known, the bleach % of the ORG stimulus was calculated to be approximately 40% [18].

### 2.2. Imaging protocol

OCT volumes were acquired in a raster-scan, BM-mode fashion, with the y-galvo disabled, and the x-galvo scanned at a single B-scan location on the retina. The B-scan settings for all experiments were 500 A-scans / B-scan (250 A-scans per spectrometer) with an 80% duty cycle (100 A-scans for flyback). The two spectrometers were configured such that the camera operation trigger is imposed alternately to each spectrometer, so that one detector operates while the other is down. In this mode, the system can acquire data with a 100% duty cycle without any integration of dead time, achieving double the effective A-scan rate compared to a single spectrometer [20].

To investigate the effect of OCT scan-speed on ORG, the camera exposure times were set to either 2.5 µs or 5.0 µs, granting A-scan rates of either 200 kHz or 400 kHz, resulting in B-scan rates of 400 Hz (low-speed) or 800 Hz (high-speed), respectively. To investigate the impact of lateral resolution on ORG, the beam diameter incident on the cornea was set via the zoom collimator within the sample arm to be either 1.6 mm (hereafter referred to as ‘low NA’) or 4.8 mm (hereafter referred to as ‘high NA’). These labels are used as relative terms to distinguish the two conditions tested in this study and do not represent absolute thresholds. Assuming an eye length of 22.2 mm and refractive index of 1.33, this corresponds to NA = 0.048 (low NA) and NA = 0.144 (high NA), with corresponding lateral resolutions of ∼10.4 µm and ∼3.5 µm respectively [23]. It should be noted that while diffraction limited resolution was likely not achieved at high NA, the difference in imaging NA permitted visualisation of the cones in these healthy young volunteers, facilitating the desired comparison of impact on the ORG signal. In all experiments, the total scan time was 3.0 s (hence, high-speed mode had double the data size of low-speed mode), with the stimulus flash delivered at the 1.5 s mark. For each experiment (e.g., low-speed, high-NA), the measurement was repeated five times to facilitate signal averaging.

### 2.3. Subjects and procedures

Seven eyes from seven healthy volunteers were imaged for this study (mean age 29.0 ± 4.1 years). This study adhered to the tenets of the Declaration of Helsinki and was approved by the local institutional review board, and written informed consent was obtained from participants. Pupil dilation via tropicamide (1%) mydriatic drops was not necessary for low-NA mode but was necessary for high-NA mode, as the larger incident beam would clip on the pupil if not administered. For each change of NA condition, the tunable variable lens within the sample arm was adjusted to ensure the sharpest OCT focus on the outer retinal layers, and the focus of the ORG stimulus image was similarly optimised; these steps compensate for any shift in accommodation state between conditions. The subject’s head was placed into a chin rest with an accompanying forehead rest, but neither a head strap nor a bite-bar was used, as it was determined early on that the physical stability of subjects in the traditional headrest was sufficient to extract a reliable ORG signal.

Before and between each ORG measurement, a 30 s dark adaptation period was implemented to allow partial recovery of cone photopigment between stimuli. Cone photopigment regeneration after a moderate bleach proceeds rapidly, with the majority of the photopic response recoverable within tens of seconds [24]. Consistent with sufficient recovery, no systematic diminution of ORG response amplitude was observed across the five repeated trials acquired at each retinal location. A fixation target (FT) projected onto the retina via the DLP was set to a dim red colour setting and offset laterally from the green ORG rectangle such that it did not overlap with the stimulated region, minimizing the possibility that the FT could bleach the imaged region before the flash. The FT was set such that the imaged region was either 2° or 9° temporal from the fovea.

### 2.4. OCT-ORG data processing

**Fig. 2.**
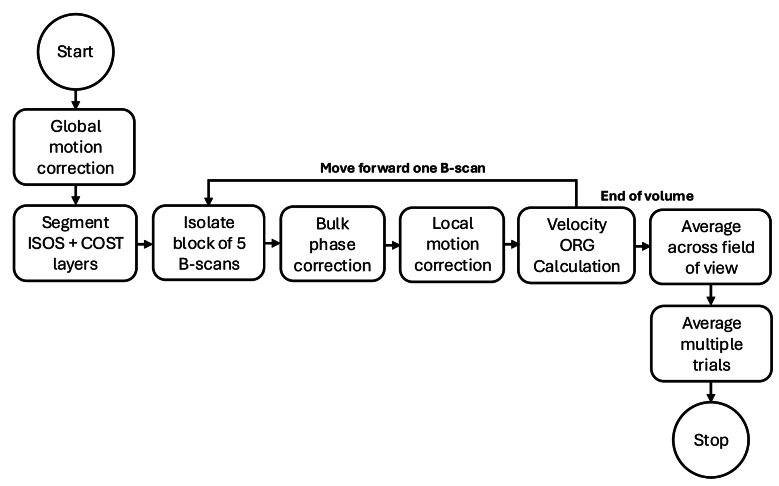
Data processing pipeline, featuring image registration, phase correction, and velocity ORG calculation.

The ORG signal-processing workflow used in this study is shown in Fig. 2. Serially acquired B-scans are first corrected for axial and lateral motion. Each B-scan is then segmented to identify the ISOS and COST (cone outer segment tips) layers. A five-frame moving window is subsequently rolled across the volume to perform local registration, ensuring precise alignment within each block. After this local registration, a bulk phase correction is applied to stabilize the pixel-wise phase within the window. Following the approach of Vienola et al. [18], the velocity ORG value at each pixel along the segmented ISOS and COST layers (and within ±1 pixel around each layer) is obtained by fitting a linear slope to the five phase values within the window. The window then advances by one B-scan, and the process repeats across the entire volume. The resulting velocity values are averaged laterally across the field of view to produce a 3.0 s ORG trace for both the ISOS and COST layers. Subtracting the COST trace from the ISOS trace yields a single velocity ORG curve representing cone outer-segment deformation. Finally, this signal is averaged across the five repeated measurements, producing one ORG curve per region of interest.

#### 2.4.1. Image registration

Robust image registration of the serially acquired B-scans is crucial to extract phase-sensitive ORG. Axial and lateral motion were corrected using a subpixel registration algorithm [25,26]. For axial correction, the estimated shift between each frame and a reference frame (initially chosen as the first frame) was applied in the spectral (k) domain: the complex OCT image was inverse-Fourier-transformed along the depth axis to recover the spectral interferogram, multiplied by a wavenumber-dependent phase ramp, and forward-transformed back to the image domain. For lateral correction, the estimated shift was applied as a phase ramp in the lateral spatial frequency domain of the OCT image — the appropriate domain for lateral shifting, since OCT lateral image formation follows standard camera optics [26]. To suppress occasional erroneous shift estimates, unusually large shifts triggered a reset of the reference frame, and the shift value was replaced with the previous estimate. This reference-reset method was used to compensate for out-of-plane motion, as such motion is not correctable when using a fixed reference frame.

#### 2.4.2. ORG signal enhancement

To improve the robustness of the ORG measurements, additional filtering was applied to restrict the analysis to pixels that contributed reliable phase information. First, each B-scan in each block of five B-scans underwent bulk phase correction to a reference frame (the first B-scan in the block). To do so, the algorithm computed the complex inner product between each frame and the reference frame, summed this product across depth, and used the resulting angle as a frame-specific global phase offset. Subtracting this offset by multiplying with a complex exponential removed slow, non-physiological phase drifts and enforced a consistent phase reference across the time series.

**Fig. 3.**
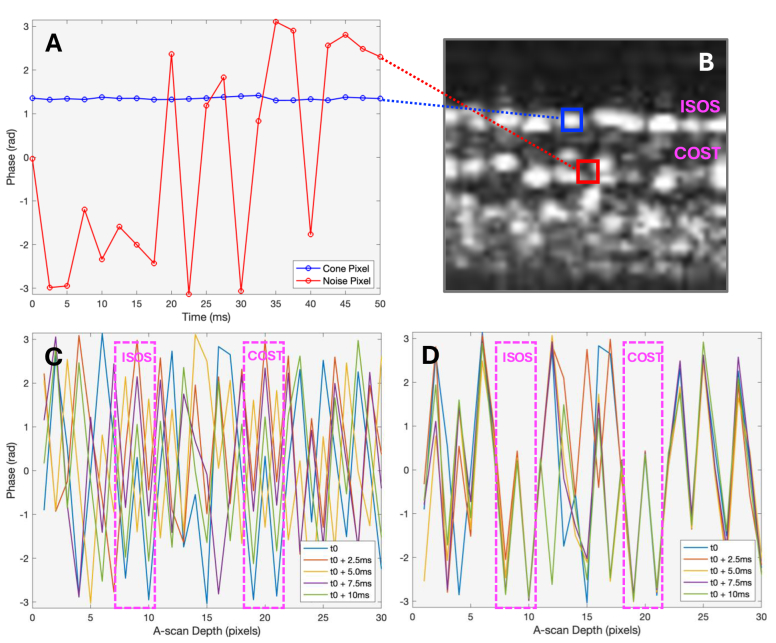
Phase signal processing for optimizing ORG. (A-B) Illustration of a high intensity cone pixel (blue) possessing stronger phase stability than a low-intensity noise pixel between cones (red). (C) An example time series of phase values from five A-scans acquired at the same retinal location. (D) The same time series after bulk phase correction, showing stabilized phase at the ISOS and COST layers.

The ISOS and COST layers were then segmented using a reflectivity-guided approach that traces each layer’s local position rather than imposing a fixed horizontal line, accounting for natural variations in outer-segment geometry across the B-scan. The average segmentation was derived from the averaged B-scan of the whole volume. Then, for each block, the segmentation was allowed to vary +/- 1 pixel axially relative to the average to account for minor motion of the subject between frames. Because OCT phase stability is strongly dependent on signal amplitude, an intensity-based threshold was applied to restrict the velocity-ORG calculation to pixels with reliable phase information. For each trial, pixels were excluded from the velocity average based on their reflectivity: an adaptive per-block threshold was derived from the intensity distribution at each retinal layer using Otsu's method for bimodal distributions or a fixed percentile fallback for unimodal ones, with thresholds smoothed temporally to ensure stable pixel inclusion across the trial. This process suppresses low-reflectivity background pixels that introduce phase noise. Figure 3 summarizes how intensity thresholding and bulk phase correction are applied to improve the fidelity of the ORG signal.

#### 2.4.3. Quantification of ORG metrics

**Fig. 4.**
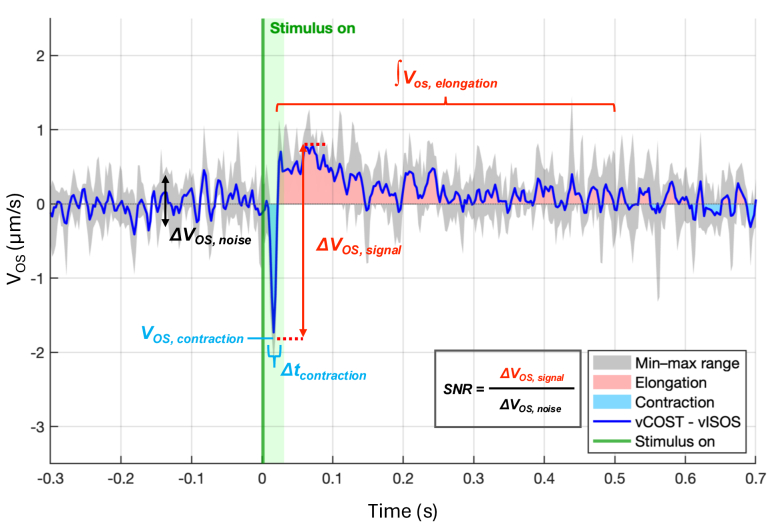
Quantification of the ORG signal, showing the key metrics: max contraction amplitude (V_OS_, _contraction_), duration of the contraction (Δt_contraction_), total elongation amount (∫V_os, elongation_), and SNR.

ORG responses were quantified using four metrics derived from the outer-segment velocity trace ( $V_{\text{OS}}$ ). Peak contraction amplitude was defined as the most negative post-stimulus value of $V_{\text{OS}}$ . Total elongation amount was computed as the time integral of $V_{\text{OS}}$ over a 500 ms interval following the contraction phase, capturing the cumulative outer-segment elongation. Contraction width was defined as the duration for which $V_{\text{OS}}$ remained below zero following stimulus onset. Finally, signal-to-noise ratio (SNR) was computed as the ratio between the ORG signal amplitude and the baseline noise. The ORG signal amplitude was quantified using a peak-to-peak metric, taken between the peak contraction value and the mean $V_{\text{OS}}$ within ±10 ms of the most positive post-stimulus value. Baseline noise was defined as the root-mean-square (RMS) of the pre-stimulus $V_{\text{OS}}$ . Figure 4 illustrates how these outcome measures were derived.

## 3. Results

Optoretinography responses were successfully obtained from all subjects across the tested acquisition conditions. In all experiments, the velocity-based ORG traces exhibited a biphasic response following stimulus onset, consisting of an initial negative velocity deflection (contraction) followed by a slower positive phase (elongation). Quantitative analysis was performed using four metrics derived from the outer-segment velocity trace: peak contraction amplitude, total elongation amount, contraction width, and signal-to-noise ratio (SNR), as defined in Section 2.4.3. For each experimental condition, metrics were computed per eye and summarized across subjects in Table 1.

**Table 1.** Summary of ORG metrics across all experimental conditions

Metric	Peak Contraction (µm/s)	Contraction Width (ms)	Total Elongation (nm)	SNR
***Lateral resolution***				
Low NA, 2° temporal	−1.72 ± 0.59	22.5 ± 14.1	118 ± 22	19.1 ± 6.8
High NA, 2° temporal	−1.68 ± 0.60	19.2 ± 7.2	112 ± 29	14.2 ± 4.4
Low NA, 9° temporal	−1.30 ± 0.13	15.8 ± 3.8	89 ± 29	14.1 ± 1.1
High NA, 9° temporal	−1.24 ± 0.09	19.2 ± 11.5	87 ± 15	9.0 ± 2.5
***Acquisition speed (low NA, 2°)***				
Low-speed (400 Hz)	−1.74 ± 0.06	15.8 ± 3.8	118 ± 19	19.8 ± 3.7
High-speed (800 Hz)	−2.33 ± 0.46	11.7 ± 6.3	108 ± 25	14.9 ± 3.9
***Inter-trial averaging (low NA, 2°, filtered)***				
Single acquisition	—	—	—	8.5 ± 2.7
5 trials averaged	—	—	—	19.1 ± 6.8
***Spatial averaging (low NA, 2°, unfiltered)***				
40 µm	—	—	—	2.6 ± 1.0
400 µm (full FOV)	—	—	—	6.5 ± 2.3

Before examining the effects of individual acquisition parameters, we first characterise the trial-to-trial repeatability of the ORG measurements across all conditions. For each eye and condition, the coefficient of variation (CV) of each metric was computed across the five repeated acquisitions. Averaged across subjects and conditions, the CV for peak contraction amplitude was 23.0 ± 5.2% and for total elongation amount was 27.0 ± 5.9%, indicating moderate inter-trial variability. Importantly, the biphasic ORG waveform — comprising both a contraction and elongation phase — was observed in all five trials across all subjects and conditions, confirming that the signal is reliably elicited under the tested acquisition parameters.

### 3.1. Impact of lateral resolution

**Fig. 5.**
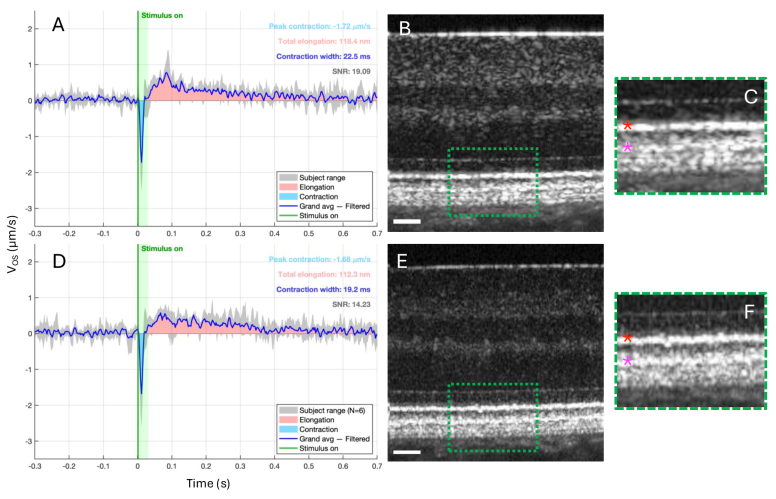
Impact of lateral resolution on ORG near the fovea. Mean ORG traces across seven eyes for low-NA (A) and high-NA (D) imaging acquired 2° temporal to the fovea. (B,E) Representative averaged B-scans from one subject at low and high NA respectively, and corresponding magnified views of the outer retinal bands are shown in (C,F). Red and magenta stars indicate the ISOS and COST layers, respectively. Scale bar = 50 µm.

The effect of lateral resolution on ORG measurements was evaluated by comparing low-NA and high-NA acquisitions in seven eyes for which matched measurements were obtained at the same retinal location. Representative structural OCT images for both conditions are shown in Fig. 5, qualitatively illustrating the increased transverse resolution and cone visibility achieved at higher NA when imaging 2° temporal to the fovea. Despite the structural differences in the OCT intensity images, the averaged ORG velocity traces were similar between low-NA and high-NA acquisitions across subjects, with low NA peak contraction amplitude, contraction width, and SNR presenting as slightly higher than their high NA counterparts. However, contraction width was found to be quite variable across subjects at this imaging location. These values are detailed in Table 1.

**Fig. 6.**
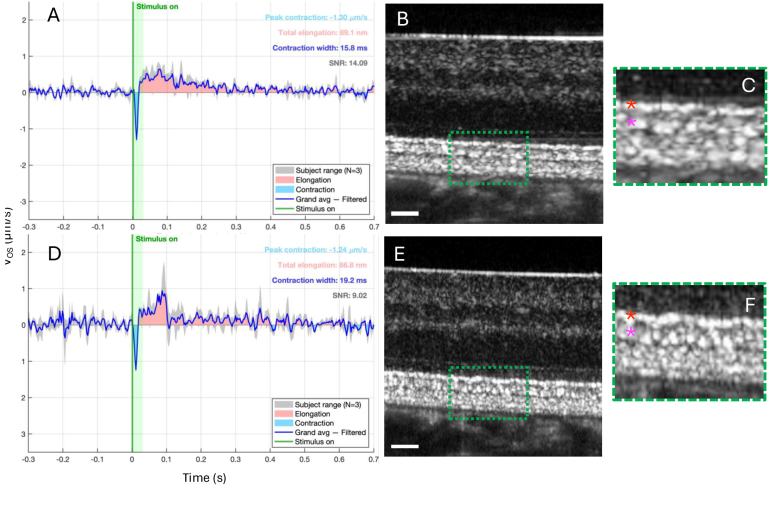
Impact of lateral resolution on ORG in the temporal parafovea. Mean ORG traces across four eyes for low-NA (A) and high-NA (D) imaging acquired 9° temporal to the fovea. (B,E) Representative averaged B-scans from one subject at low and high NA respectively, and corresponding magnified views of the outer retinal bands are shown in (C,F). Red and magenta stars indicate the ISOS and COST layers, respectively. Scale bar = 50 µm.

A smaller subset of four eyes was imaged 9° temporal to the fovea in both low and high NA modes. This enabled additional comparison at the level of individual cone visibility, as illustrated in Fig. 6. While higher NA improved cone contrast in the structural images, increased phase variability was observed in low-reflectivity pixels between cones, necessitating more aggressive intensity-based filtering. This effect was not present in low-NA data, where the reduced spatial sampling resulted in fewer unstable phase pixels contributing to the averaged ORG trace. As expected, the ORG signal magnitudes decreased with increased distance from the fovea. At 9° temporal, the peak contraction amplitude at low NA was −1.30 ± 0.13 µm/s, compared to −1.24 ± 0.09 µm/s at high NA. The total elongation amount was 89 ± 29 nm for low NA and 87 ± 15 nm for high NA. The contraction width was 15.8 ± 3.8 ms at low NA and 19.2 ± 11.5 ms at high NA.

**Fig. 7.**
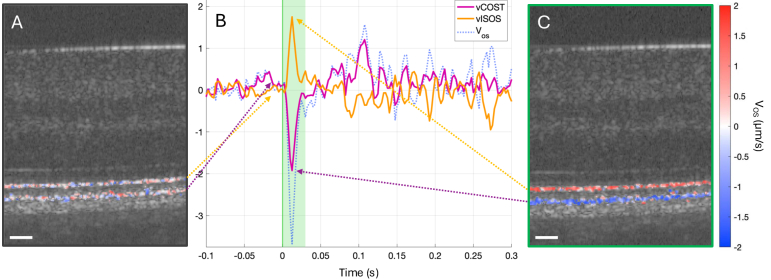
Pixel-wise spatial localization of the ORG signal. By calculating the velocity ORG curves for the ISOS and COST layers separately, we can identify which pixels in each layer contribute to the resulting averaged curve. A) Baseline ORG pseudo-colour image extracted immediately before the stimulus. B) Averaged velocity ORG curve averaged across the field of view, showing the V_COST_ (magenta), V_ISOS_ (yellow), and V_OS_ (dashed blue) curves independently. C) ORG pseudo-colour image extracted at the time point of maximum contraction. Red-shifted pixels denote positive (downward) velocity, while blue-shifted pixels denote negative (upward) velocity.

Figure 7 demonstrates how the ORG signals from the ISOS and COST layers can be delineated. Plotting the layer-specific velocity traces separately confirms that V_ISOS_ and V_COST_ display distinct temporal dynamics which, when subtracted from each other, result in the net outer-segment velocity (V_OS_) (Fig. 7(B)). By calculating and visualizing the velocity at each segmented pixel prior to lateral averaging, we can identify the spatial distribution of phase changes that contribute to the composite outer-segment velocity curve. The baseline pseudo-colour image (Fig. 7(A)) demonstrates minimal velocity signal prior to stimulation, whereas at the time of peak contraction (Fig. 7(C)), a subset of pixels along the segmented outer retinal bands exhibits coherent velocity shifts.

### 3.2. Impact of acquisition speed

**Fig. 8.**
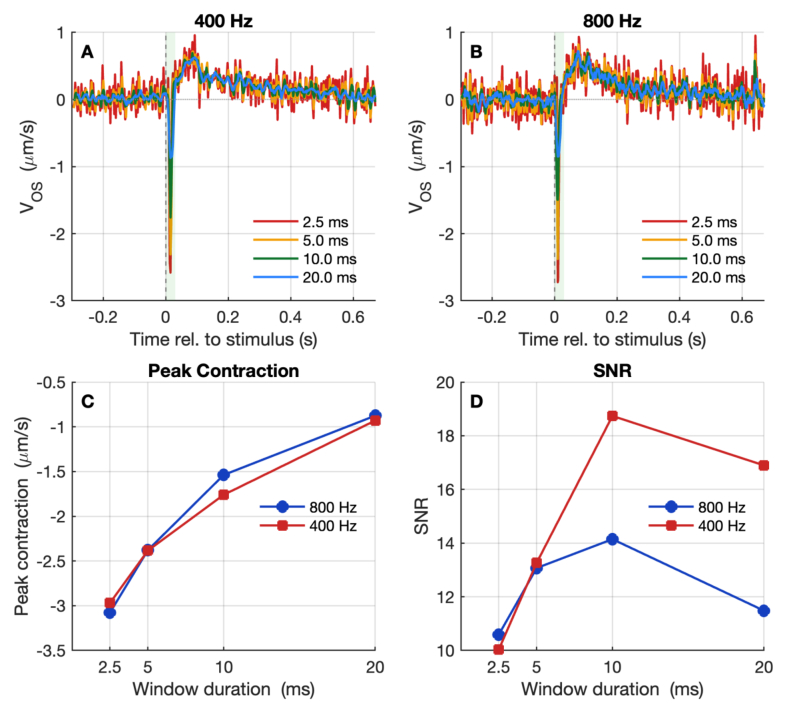
Impact of velocity estimation window duration and acquisition speed on ORG metrics. Mean ORG velocity traces averaged across three subjects at 400 Hz (A) and 800 Hz (B) for four velocity window durations: 2.5 ms (red), 5.0 ms (orange), 10.0 ms (green), and 20.0 ms (blue). The stimulus flash onset is indicated by the dashed vertical line. (C) Peak contraction amplitude and (D) SNR as functions of velocity window duration for both acquisition speeds.

The influence of acquisition speed was assessed by comparing ORG measurements obtained at A-scan rates of 200 kHz and 400 kHz, corresponding to B-scan rates of 400 Hz (low-speed mode) and 800 Hz (high-speed mode), respectively. All other acquisition parameters, including stimulus strength, lateral resolution, and total acquisition duration, were held constant. Three eyes from three subjects were imaged for this experiment. To disentangle the effects of acquisition speed from those of the velocity estimation window duration, both the 400 Hz and 800 Hz datasets were analysed across four temporally matched velocity window durations: 2.5, 5, 10, and 20 ms. Results are summarised in Fig. 8.

Peak contraction amplitude and contraction width were found to be almost entirely determined by the velocity window duration, with 400 Hz and 800 Hz producing near-identical values at each matched window size. At 2.5 ms, both conditions yielded peak contraction amplitudes of approximately −3.0 µm/s, converging to approximately −0.95 µm/s at 20 ms, with contraction width scaling correspondingly from ∼5 ms to ∼22 ms. SNR, by contrast, showed a genuine acquisition-speed dependence. At matched window durations of 10 ms and above, 400 Hz outperformed 800 Hz in SNR (18.8 vs 14.1 at 10 ms; 16.9 vs 11.5 at 20 ms). Both conditions achieved peak SNR at a 10 ms window, with SNR declining at 20 ms due to the flattening of the contraction peak. Elongation amount showed a similar trend, with 400 Hz yielding slightly higher values (∼120 nm vs ∼115 nm) at window sizes of 10 ms and above.

These results indicate that for this system, 400 Hz B-scan acquisition rate preserves a modest but consistent SNR advantage over 800 Hz at equivalent temporal resolutions, attributed to the longer per-frame exposure time and consequently lower phase noise floor.

### 3.3. Impact of spatial and trial averaging

**Fig. 9.**
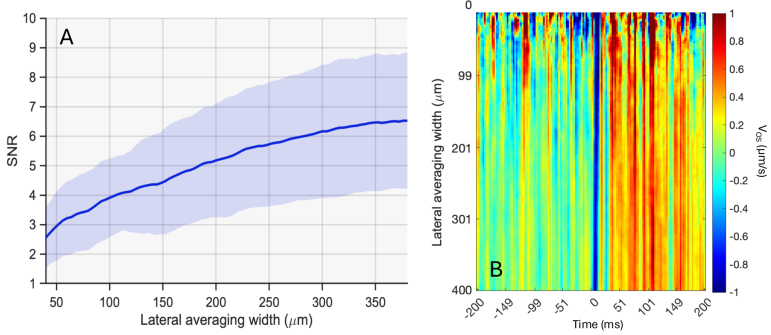
Impact of spatial averaging on ORG. A) SNR as a function of lateral averaging for unfiltered single ORG measurements, summarized across subjects in the low NA 2° condition. B) Velocity ORG signal as a function of lateral averaging for one subject, where blue represents negative ORG velocity (contraction), while red represents positive ORG velocity (elongation).

The effect of averaging on ORG signal quality was evaluated by varying both the number of repeated measurements per eye (trial averaging) and the degree of lateral averaging across the imaged field of view (spatial averaging). In general, increasing spatial averaging led to a progressive improvement in SNR up to a plateau at ∼300 µm of averaging. Across subjects, SNR increased from 2.6 ± 1.0 with 40 µm of spatial averaging to 6.5 ± 2.3 when averaging the full 400 µm field of view on unfiltered ORG measurements. This calculation was done on unfiltered data to remove the influence of the intensity-based filter mask. Figure 9 summarizes the impact of spatial averaging on the ORG signal and its SNR.

Similar trends were observed for trial averaging: averaging across multiple repeated acquisitions reduced the effect of inter-trial variability and reduced baseline noise, resulting in improved trace stability. For example, for the low NA imaging condition, SNR increased from 8.5 ± 2.7 for single acquisitions to 19.1 ± 6.8 when averaging 5 filtered measurements.

### 3.4. Impact of intensity filtering

The effect of the adaptive intensity-based filtering step on ORG signal quality was evaluated by comparing unfiltered and filtered ORG metrics across NA conditions and retinal eccentricities. As described in Section 2.4.2, this step excludes low-reflectivity pixels whose phase measurements are dominated by noise rather than physiological signal, using an adaptive per-block threshold derived from the layer intensity distribution. Table 2 summarises the results, and demonstrates that filtering consistently and substantially improved all three ORG metrics across every tested condition, with SNR showing the largest absolute benefit.

**Table 2.** Impact of intensity filtering on ORG metrics across NA and eccentricity conditions. Values are mean ± SD across subjects

Condition	Peak Contraction (µm/s)		Total Elongation (nm)		SNR	
	Unfiltered	Filtered	Unfiltered	Filtered	Unfiltered	Filtered
Low NA, 2°	−1.56 ± 0.56	−1.72 ± 0.59	108 ± 27	118 ± 22	14.8 ± 4.9	19.1 ± 6.8
High NA, 2°	−1.47 ± 0.50	−1.68 ± 0.60	96 ± 29	112 ± 29	11.5 ± 3.5	14.2 ± 4.4
Low NA, 9°	−1.12 ± 0.13	−1.30 ± 0.14	88 ± 21	89 ± 30	11.1 ± 1.5	14.1 ± 1.1
High NA, 9°	−0.95 ± 0.40	−1.24 ± 0.09	73 ± 12	87 ± 15	7.1 ± 2.2	9.0 ± 2.5

At 2° temporal, filtering improved SNR from 14.8 ± 4.9 to 19.1 ± 6.8 at low NA, and from 11.5 ± 3.5 to 14.2 ± 4.4 at high NA. The benefit was similarly present at 9° temporal, where SNR increased from 11.1 ± 1.5 to 14.1 ± 1.1 at low NA, and from 7.1 ± 2.2 to 9.0 ± 2.5 at high NA. Notably, ORG metrics were consistently lower for high NA than their low NA counterparts, and this disparity was most pronounced at 9° temporal. The application of the intensity filter to the high NA data mostly recovered the amplitude of the contraction and elongation signals, but with a noticeably reduced SNR. This is partly attributable to the more demanding filtering requirements at high NA: the tighter focal spot resolves inter-cone dark regions more sharply, increasing the proportion of low-reflectivity pixels excluded by the adaptive threshold. Indeed, on average, 23% fewer pixels passed the filter in the high NA condition compared to low NA, reducing the number of pixels contributing to the spatial average and thereby limiting the averaging gain on SNR.

## 4. Discussion

In this study, we investigated how key acquisition parameters influence the fidelity of phase-based optoretinography when implemented on a raster-scanning spectral-domain OCT platform. The motivation was pragmatic: to assess requirements in terms of lateral resolution, scan speed, and averaging while still obtaining a detectable and repeatable ORG signal using hardware configurations that resemble those already deployed in clinical OCT systems. The focus was on identifying how clinically realistic acquisition parameters impact the canonical ORG features.

Across all acquisition conditions tested, a biphasic ORG response consisting of a rapid contraction followed by a slower elongation was consistently observed, in agreement with prior phase-based ORG studies. This indicates that the velocity-based processing framework is sufficiently robust to extract physiologically meaningful signals even when spatial resolution and acquisition speed are reduced relative to many research-grade ORG systems. Importantly, these signals were obtained without resolving individual cones and without requiring ultrahigh B-scan rates.

### 4.1. Impact of lateral resolution and filtering

Varying the numerical aperture revealed relatively small differences in the extracted ORG metrics when signals were averaged across the field of view. While higher NA improved the apparent sharpness of outer retinal layers, this did not translate into a proportional improvement in contraction amplitude, elongation amount, or contraction width. In fact, in most cases, lower NA acquisitions produced more stable ORG traces when appropriate intensity-based filtering was not applied. This likely reflects the fact that higher NA images contain a greater proportion of low-reflectivity pixels between cones, which exhibit reduced phase stability — a problem that is compounded at higher eccentricities where inter-cone dark regions are more prominent.

The consistent benefit of adaptive intensity filtering across all conditions therefore underscores the importance of pixel selection in velocity-based ORG: because phase stability scales with reflectivity, unfiltered averages are corrupted by low-SNR pixels whose phase measurements contribute noise rather than coherent phase signal. After filtering, high-NA ORG metrics more closely resemble their low-NA counterparts, supporting the interpretation that the modest amplitude differences reflect reduced pixel yield rather than a weaker photoreceptor response. The residual SNR gap between high and low NA conditions after filtering is consistent with this view: on average, 23% fewer pixels passed the intensity threshold in the high NA condition, reducing the spatial averaging gain.

Altogether, these findings suggest that for clinical applications where the interest lies in the ensemble response of dozens of cones rather than cellular-level ORG, higher lateral resolution may not be necessary and a lower-resolution image may even be preferable — particularly given that lower NA imaging reduces the need for pupil dilation and relaxes alignment constraints, both of which improve patient comfort and workflow compatibility. However, it should be noted that this conclusion is specific to averaged ORG metrics and does not negate the value of high-resolution imaging for applications that require cellular-scale functional mapping.

Finally, it should be noted that the conclusions regarding imaging NA drawn here are specific to fibre-based point-scanning OCT, in which the single-mode fibre inherently couples the illumination and detection paths such that input and output NAs are matched. In free-space, line-scan, or full-field OCT modalities, illumination and detection NA can be controlled independently, and the relationship between beam diameter and ORG signal quality may differ accordingly.

### 4.2. Impact of acquisition speed

The velocity window duration emerged as the primary factor governing contraction amplitude and width, with acquisition speed having negligible independent influence on these metrics when window duration is held constant. The more relevant finding is the SNR advantage of 400 Hz over 800 Hz at matched window sizes of 10 ms or longer, which we attribute to the longer per-frame camera exposure (5.0 µs vs 2.5 µs) reducing the phase noise floor. This suggests that for averaged ORG metrics, moderate acquisition speeds may be sufficient to capture the essential response. However, we recognize that only two acquisition speeds were analysed here, and previous studies using faster acquisition rates have been conducted and have their own strengths. It is possible that a combination of scan patterns and speeds may be ideal to sample the initial contractile response more densely in time, followed by a slower acquisition over a wider field of view to capture the subsequent elongation.

### 4.3. Impact of spatial and trial averaging

Both spatial and trial averaging improved ORG SNR. Spatial averaging across the imaged field of view proved effective at suppressing local phase noise, reinforcing the notion that robust ORG signals can be obtained without isolating individual photoreceptors. For spatial averaging, the improvement in SNR was lower than the theoretical limit of √N, which likely reflects the correlated nature of adjacent samples within a B-scan, and the plateau at ∼300 µm represents the effective spatial correlation length beyond which additional averaging yields diminishing returns. Meanwhile, trial averaging improved SNR in close agreement with the √N independent-sample prediction, suggesting that the noise in successive acquisitions is effectively uncorrelated. From a workflow standpoint, these results suggest that a small number of repeated measurements performed over a few minutes per eye may be sufficient to obtain stable ORG metrics. This aligns with the goal of keeping ORG acquisitions compatible with routine clinical imaging sessions, rather than prolonged research-style protocols.

### 4.4. Methodological considerations and limitations

Several limitations of this study should be acknowledged. First, the cohort consisted solely of young, healthy volunteers, and the parameter dependencies reported here may differ in eyes with retinal pathology, where reflectivity, phase stability, and motion characteristics are altered. Additionally, stimulus-induced pupil constriction, although not observed to cause beam clipping in this cohort, may be a more significant concern in older or pathological eyes with reduced pupil dilation. Second, the study focused on velocity-based ORG metrics; other phase- or intensity-based approaches may exhibit different sensitivities to acquisition parameters. Third, although the stimulus parameters were held constant in most experiments, stimulus strength and temporal profile are known to influence ORG dynamics and were not systematically explored here. Flicker-based stimulation paradigms, in particular, may impose different requirements on acquisition speed and averaging, and warrant separate investigation. Fourth, out-of-plane motion represents a fundamental limitation of the BM-mode B-scan ORG approach. While the registration pipeline resets the reference frame upon detecting large decorrelation events, and the short velocity-estimation windows limit the temporal propagation of motion-induced phase artefacts, no explicit frame-rejection step based on decorrelation metrics was implemented. Future implementations may benefit from such a step to further suppress motion-corrupted acquisitions.

Taken together, these results support the feasibility of performing phase-based ORG on raster-scanning OCT systems with acquisition parameters similar to those of modern clinical instruments. While such systems do not match the performance of specialized research platforms, this report demonstrates that they are capable of capturing the core functional signatures of photoreceptor outer-segment deformation when appropriate processing and averaging strategies are employed.

## 5. Conclusion

This work outlines a set of practical trade-offs that can inform system design and protocol development for proto-clinical ORG studies. Future work will be required to determine how these findings generalize to patient populations and whether specific acquisition regimes are better suited to detecting early functional deficits in retinal disease.

## Data Availability

Data underlying the results presented in this paper are not publicly available at this time but may be obtained from the authors upon reasonable request.
